# Somatic embryogenesis in ferns: a new experimental system

**DOI:** 10.1007/s00299-015-1741-9

**Published:** 2015-01-20

**Authors:** Anna Mikuła, Mariusz Pożoga, Karolina Tomiczak, Jan J. Rybczyński

**Affiliations:** Polish Academy of Sciences Botanical Garden, Center for Biological Diversity Conservation in Powsin, Prawdziwka 2, 02-973 Warsaw, Poland

**Keywords:** *Cyathea delgadii*, Hormone-free medium, Light microscopy, Methyl salicylate, Somatic embryo, Tree fern

## Abstract

*****Key message***:**

**Somatic embryogenesis has never been reported in ferns. The study showed that it is much easier to evoke the acquisition and expression of embryogenic competence in ferns than in spermatophytes.**

**Abstract:**

We discovered that the tree fern *Cyathea delgadii* offers an effective model for the reproducible and rapid formation of somatic embryos on hormone-free medium. Our study provides cyto-morphological evidence for the single cell origin and development of somatic embryos. Somatic embryogenesis (SE) in both primary and secondary explants was induced on half-strength micro- and macro-nutrients Murashige and Skoog medium without the application of exogenous plant growth regulators, in darkness. The early stage of SE was characterized by sequential perpendicular cell divisions of an individual epidermal cell of etiolated stipe explant. These resulted in the formation of a linear pro-embryo. Later their development resembled that of the zygotic embryo. We defined three morphogenetic stages of fern somatic embryo development: linear, early and late embryonic leaf stage. The transition from somatic embryo to juvenile sporophyte was quick and proceeded without interruption caused by dormancy. Following 9 weeks of culture the efficiency of somatic embryogenesis reached 12–13 embryos per responding explant. Spontaneous formation of somatic embryos and callus production, which improved the effectiveness of the process sevenfold in 10-month-long culture, occurred without subculturing. The tendency for *C. delgadii* to propagate by SE in vitro makes this species an excellent model for studies relating to asexual embryogenesis and the endogenous hormonal regulation of that process and opens new avenues of experimentation.

## Introduction

Somatic embryogenesis is the developmental pathway by which plant somatic cells develop into somatic embryos. Apart from the lack of syngamy, these resemble zygotic embryos. SE is currently used to propagate hundreds of species of seed plant in vitro, and forms the basis of fundamental studies that help us understand how a single somatic cell develops to form an entire plant (Vogel 2005). The process serves as a model system for the study of molecular, biochemical and physiological events that occur during both the induction and the development of the embryo. Although SE was discovered more than half a century ago (Steward et al. 1958), our entire knowledge of this process is based on seed plants. It has not previously been reported for monilophytes. Moreover, it has been reported only twice for cryptogams, and in each case these were lycopods, i.e. *Lycopodiella inundata* (L.) Holub (Atmane et al. 2000) and *Huperzia selago* (L.) Bernh. Ex Schrank and Mart. [*Lycopodium selago* L.] (Szypuła et al. 2005). Since ferns and spermatophytes are sister lineages (Pryer et al. 2001), ferns are useful subjects for developmental and morphological research, comparative evolution, and functional genomics (Johnson and Renzaglia 2008, 2009; Der et al. 2011; Tomescu 2011; Vasco et al. 2013). They can also help to improve our understanding of SE.

In terms of zygotic embryogenesis, ferns have been relatively under-investigated compared to spermatophytes. Extensive studies of the sexual reproduction of ferns were conducted at the turn of the twentieth century and a considerable amount of information regarding the embryology of this plant group was collected, collated and published by Wardlaw (1955). He demonstrated the pattern of development that occurred during the early embryology of various fern species and how the two-celled early embryo of leptosporangiate ferns underwent six additional regular divisions to create an eight-celled embryo. The proliferation of zygotic initial cells eventually led to a four-part embryo, the four quadrants forming the root, leaf, foot, and shoot apex, respectively, that would later produce additional vegetative organs. Subsequently, morphological, cytological, and biochemical studies conducted on fern zygotic embryos at various stages of development were summarized by DeMaggo (1977). Since that time, progress has not been as rapid or as extensive in its scope, and there still remains a dearth of embryological information relating to those processes that follow on from cell and organ differentiation. By contrast, there is a wealth of information available relating to the developmental anatomy and morphology of shoot, leaf and root of the fern sporophyte (White and Turner 1995). Currently, researchers aim for a better understanding of the evolution of land plants, and alternation of generations, as observed in ferns (Niklas and Kutschera 2009; Ligrone et al. 2012). Johnson and Renzaglia (2008, 2009) elucidated the development of the embryo and gametophyte placenta for the fern model *Ceratopteris richardii* Brongn. and significantly broadened our knowledge of the development of the fern zygotic embryo. It was also shown that auxin is involved in the initial zygotic cell division of *Marsilea vestita* Hook. & Grev. and organization of body plan in fern sporophytes (Poli 2005).

In addition to the sexual life cycle, some fern species undergo asexual development resulting in the formation of sporophytes from gametophyte somatic cells (apogamy) (Raghavan 1989). Either obligate or induced apogamy is considered equivalent to organogenesis of sporophytes (Raghavan 1989; Fernández et al. 1996; Gabancho et al. 2010). An apogamous system of reproduction occurs both in nature and in vitro, and it has been regularly studied ever since its discovery by Farlow (1874) up to the present time (Cordle et al. 2011). This method of reproduction became established in fern lineages that experienced frequent reticulate evolution in combination with polyploidy and has been recognized for approximately 10 % of extant ferns (Liu et al. 2012). However, apogamy has been only sporadically reported for tree fern species (Stokey 1918; Parajuli and Joshi 2014).

Propagation of tree fern species is a difficult challenge and thus, few tree ferns are propagated by commercial nurseries. Some of the species can be propagated by spores, but they cannot be propagated vegetatively as they do not produce offsets from their ‘trunk’ (Large and Braggins 2004). However, for commercial purposes, the erect ‘trunk’ of tree ferns (e.g. *Dicksonia antarctica* Labill.) can be cut, transported and replanted, and can still continue to grow, as long as the crown remains intact (FPA Biodiversity Program 2012). Over the last few years, the propagation of tree fern species from spores has become the priority area for ex situ conservation studies (Simabukuro et al. 1998a, b; Arens 2001; Hiendlmeyer and Randi 2007; Rechenmacher et al. 2010; Ranil et al. 2011; Martíez et al. 2014). Conversely, methods of in vitro culture have been exploited for the propagation of tree ferns by gametophyte multiplication and sporophyte regeneration. Media used for growth and proliferation of gametophytes were either supplemented with plant growth regulators (PGRs) (Bonomo et al. 2013; Das et al. 2013) or not (Goller and Rybczyński 1995, 2007; Kuriyama et al. 2004; Khare et al. 2005; Moura et al. 2012). The application of biotechnology methods for tree ferns was summarized by Rybczyński and Mikuła (2011). Their list of 20 species has recently been extended by at least 3 new taxa, namely: *Alsophila odonelliana* (Alston) Lehnert*, Cyathea gigantea* (Wall. ex. Hook.) and *C. cunninghamii* Hook. f. (Moura et al. 2012; Bonomo et al. 2013; Das et al. 2013). Gametophytes cultured in vitro also provide sufficient plant material for cryo-studies and the long-term preservation of tree ferns in liquid nitrogen (Mikuła et al. 2011a). Further methods are required for the efficient, quick and effective propagation of tree fern species in vitro.

The present work relates to *Cyathea delgadii* Sternb., a species of evergreen, non-seasonal tree fern (10 m tall) native to the gallery, montane, cloud, and rain forests of the Caribbean, Central and South America, including valuable ecoregions of the Atlantic Forest. It grows at an elevation of 100–2,730 m above sea level (Oliveira-Filho and Ratter 1995). *Cyathea delgadii* is a member of a large complex centered on *Cyathea fulva* (M. Martens & Galeotti) Fée (Arens 2001). It produces spores all the year round (some 300 million spores per frond), but their viability, like the spores of most species belonging to family Cyatheaceae, diminishes after a few weeks of storage at room temperature or after a 2-year period of storage at 4 °C (Simabukuro et al. 1998a). This species is cultivated as a garden ornamental plant (Hiendlmeyer and Randi 2007).

Our study focuses on the induction and description of SE in the fern *C. delgadii*, belonging to a group of plants (Monilophyta) for which this phenomenon has not yet been reported. Emphasis is focused on cyto-morphological evidence for SE induction, embryo development, and the efficiency of this process during short- and long-term culture.

## Materials and methods

### Plant material

Laminae of *C. delgadii* fronds were collected from a plant growing in the greenhouse of the PAS Botanical Garden—CBDC, Warsaw, Poland. They were dried at room temperature for 5 days to liberate spores. The released spores were surface sterilized by wrapping sporangia in Whatman no. 1 filter paper and immersing the package in 70 % (v/v) ethanol for 30 s and in 5 % (v/v) commercial bleach (Domestos) for 20 min and then washing the package three times in sterile distilled water. After disinfection, spores were blotted onto medium containing half-strength MS micro- and macro-nutrients (Murashige and Skoog 1962) with full complement of vitamins (1/2MS), 2 % (w/v) sucrose and 0.7 % (w/v) plant agar. The pH of the medium was adjusted to 5.8 before autoclaving at 120 °C for 20 min. The spores germinated at 22 ± 1 °C under a 16/8 h photoperiod, at a light intensity of 3.5 μE m^−2 ^s^−1^. The young gametophytes were transferred separately onto fresh 1/2MS medium and subcultured only once. They were maintained in subculture until they reached maturity. After spontaneous syngamy, zygotic embryos and young sporophytes were produced after 1 year of gametophyte culture.

### Somatic embryogenesis induction and the assessment of its efficiency

**Fig. 1:**
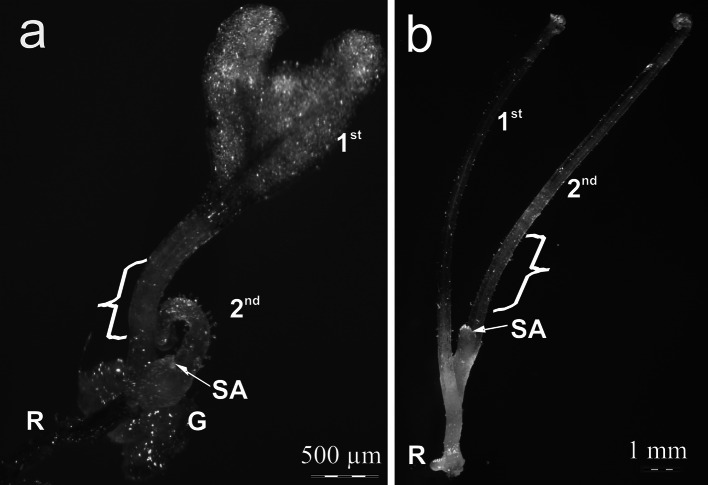
Details of origin of plant material used for culture initiation. **a** A young zygotic embryo-derived sporophyte showing the first two fronds, which were used as a source of primary explants. **b** A young somatic embryo-derived etiolated sporophyte showing the first two fronds, which were used as a source of secondary explants. *Bracket* shows an initial explant. *G* gametophyte, *R* adventitious root, *SA* shoot apex, *1st* first frond, *2nd* second frond

Stipes of zygotic embryo-derived sporophytes (Fig. 1a) developed under 16/8 h photoperiod were used for the initiation of primary SE. Secondary SE was induced on intact somatic embryos that had reached the first crozier stage and on the stipes of somatic embryo-derived sporophytes (Fig. 1b) that had developed 2 or 3 fronds, growing in darkness. The plant material was cultured on 1/2MS agar medium supplemented with 2 % (w/v) sucrose and 0.7 % (w/v) plant agar. The cultures were kept in a climatic chamber at +22 ± 2 °C, in constant darkness, and at a relative humidity of 35–55 %.

The percentage of responding explants and the number of somatic embryos per responding explant was calculated after 9 weeks of culture. Subsequent evaluations were carried out every month for almost 1 year of culture. The somatic embryo production capacity index (SEPCI) was calculated by multiplying the proliferation percentage by the number of somatic embryos formed per explant and then dividing the result by 100.

### Sporophyte acclimatization and transfer to soil

For acclimatization, sporophytes cultured under 16/8 h photoperiod conditions were used. Sporophytes with 4–6 fronds were transferred to pots of peat and perlite (3:1) substrate, and kept in mini greenhouses. The potting mixture was autoclaved at 121 °C for 20 min. The mini greenhouses were lightly ventilated daily, and the sporophytes periodically misted for 4-6 weeks. The plantlets were then transferred to a greenhouse.

### Microscopic preparation

Stipes of somatic embryo-derived sporophytes were fixed in 2 % glutaraldehyde in 0.1 M cacodylate buffer (pH 7.2) at room temperature for 2 h, rinsed three times with 0.1 M cacodylate buffer (pH 7.2), and post-fixed with 2 % OsO_4_ (osmium tetroxide) at 4 °C overnight. After rinsing in 0.1 M cacodylate buffer, explants were dehydrated in a graded ethanol series (30 → 50 → 70 → 90 → 96 → 99.8 %), followed by mixtures of absolute ethanol and propylene oxide (3:1; 1:1; 1:3), and finally, propylene oxide. Explants were then embedded in Epon-Spurr epoxy resin mixture. Two-micrometre-thick sections were cut using an LKB ultramicrotome (Sweden) and stained for several seconds with aqueous 0.1 % toluidine blue solution. They were examined using a Vanox epifluorescence microscope (Olympus, Japan) equipped with a computer image analysis system (cellSens Standard ver. 1.7). To detect the natural red autofluorescence of chlorophyll, a blue-violet light (BV filter: 400–440 nm) was used.

For examination of the first few divisions of epidermal cells, plant material was fixed in FAA (5 parts formaldehyde:5 parts glacial acetic acid:90 parts ethanol) overnight and then dehydrated in graded ethanol solutions (70, 80 and 100 %). Next, explants were cleared in methyl salicylate as described by Young et al. (1979) and examined under a Vanox microscope (Olympus, Japan) using blue-violet light (BV filter: 400–440 nm).

### Statistical analysis

Results were analyzed by means of a one-way ANOVA analysis of variance and Fisher’s least significant difference (LSD) procedure using Statgraphics Plus software. Significance was set at the 0.05 level. The results were expressed as the mean ± standard deviation based on three independent experiments, each consisting of at least 30 explants.

## Results

### Production of zygotic embryo-derived sporophytes as a source of primary explants

Propagated gametophytes of *C. delgadii* achieved maturity within a year of in vitro culture on 1/2MS medium. Further details of sex organ formation for the species were provided by Rybczyński and Mikuła (2011). Following fertilization, the early development of the zygotic embryo was confined to the archegonium. Within 4–5 weeks, the main organographic regions had been determined. Subsequently, the first leaf elongated and the second developed (Fig. 1a). The stipes of the first young fronds, grown at a photoperiod of 16/8 h, were used for the following experiments (Fig. 1a).

### Cyto-morphological evidence for somatic embryogenesis induction and embryo development

**Fig. 2:**
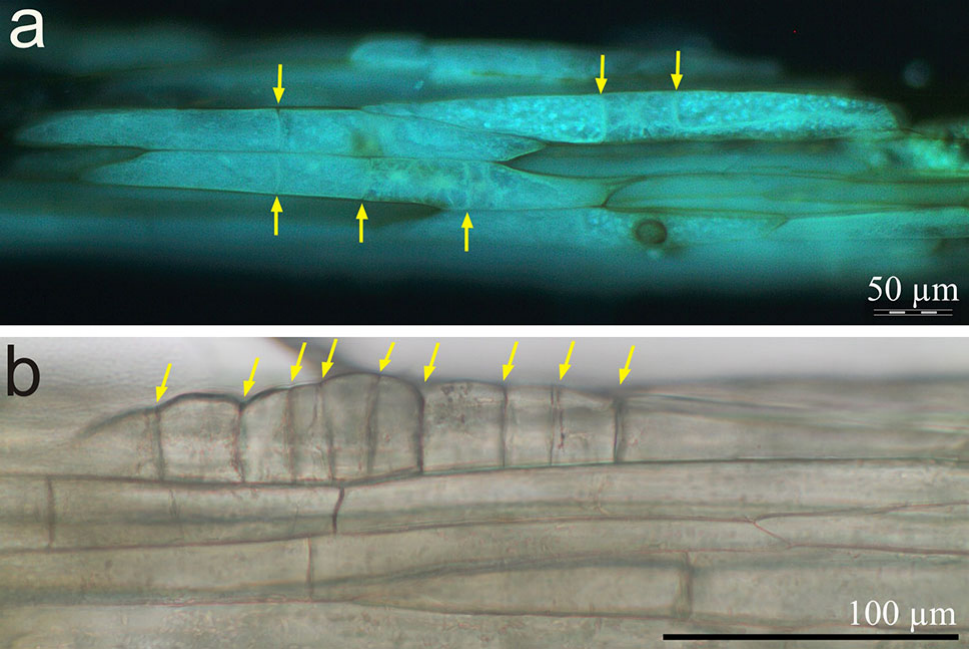
Early epidermal cell divisions of stipe explants cultured on hormone-free medium in darkness (explant cleared in methyl salicylate). **a** Three epidermal cells after one, two or three cell divisions perpendicular to the polar axis of the stipe following 2 weeks culture (BV filter: 400–440 nm). **b** Ten-celled linear somatic pro-embryo originating from a single epidermal cell of stipe explant. *Yellow arrows* show cell walls (color figure online)

Within 2 weeks following culture initiation, divisions of epidermal cells of stipe explants began (Fig. 2a). The first division of the epidermal cell was perpendicular to the polar axis of the explant and led to the formation of two, almost equal or unequal daughter cells. The next few divisions (divisions 8–10) were also perpendicular to the stipe axis (Fig. 2b). Within 3 weeks of culture, these divisions resulted in the formation of linear somatic pro-embryos.

**Fig. 3:**
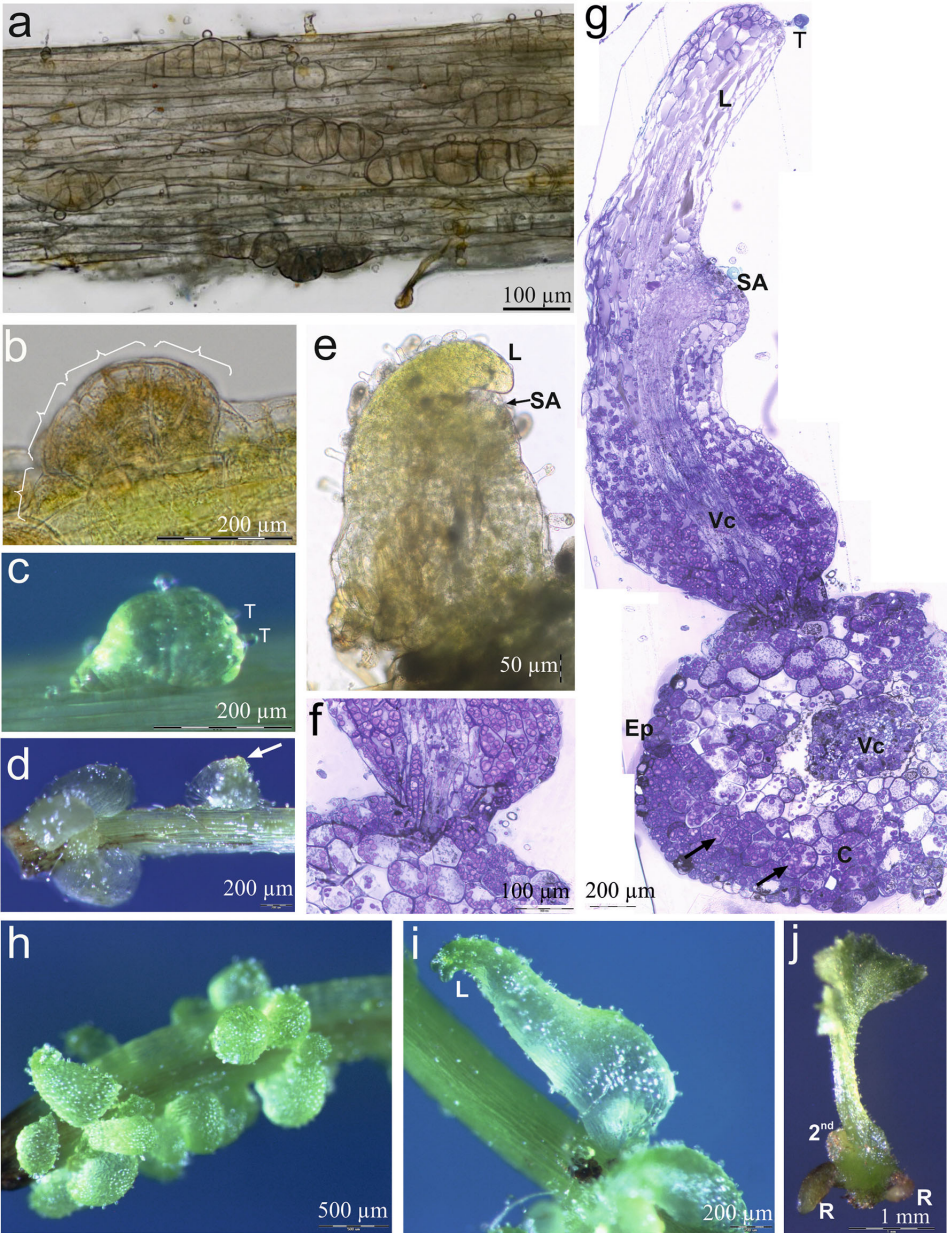
Light microscopy evidence for somatic embryo origin and development in *Cyathea delgadii*. **a** Numerous pro-embryos following several anticlinal, periclinal and inclined cell divisions of single epidermal cells of stipe explant cultured on hormone-free medium, in darkness. **b** Four-segmented somatic pro-embryo developed directly on the surface of stipe. **c** Trichomes located on one side of immature, multicellular somatic embryo. **d** Differentiation of the embryonic leaf (*arrow*). **e** A somatic embryo showing the first leaf and primordium of the second leaf (squashed specimen). **f** Junction between somatic embryo and initial stipe explant showing epidermal origin of embryo (semi-thin section stained with toluidine blue). **g** Longitudinal section of well-developed somatic embryo showing the first leaf, shoot apex and primordium of the second leaf, as well as transverse section of the stipe explant; 6 weeks of culture (*arrows* indicate amyloplasts). **h** Numerous somatic embryos with first leaf after 6 weeks growth. **i** Partly *green*, differentiated lamina of the first leaf of juvenile sporophyte. **j** Somatic embryo-derived young sporophyte showing extended lamina of primary frond and primordium of second leaf, as well as two roots, following development in the presence of light. *C* cortex, *Ep* epidermal cells, *L* first leaf, *R* root, *SA* shoot apex, *T* trichomes, *Vc* axial cylinder (color figure online)

Dividing epidermal cells were widely distributed along the explants (Fig. 3a). Within 3 weeks following culture initiation, numerous epidermal cells of stipe explants were present, resulting from several anticlinal, periclinal and inclined cell divisions. These irregular divisions led to the formation of four separate and unequally sized segments of the pro-embryo (Fig. 3b). During the early stage of somatic embryo development, trichomes developed on two of four of the visible segments of the pro-embryo (Fig. 3c). Further development of somatic embryos focused on the differentiation of the embryonic leaf (Fig. 3d). Later, the emerging lamina primordium of the first frond and primordium of the shoot apex were visible (Fig. 3e).

Histological analysis revealed that the somatic embryos originated directly from epidermal cells of stipe explants (Fig. 3f, g). The structure of the stipe was maintained for the entire duration of the initial culture on 1/2MS medium. The single-layered epidermis, cortex and closely arranged axial cylinder cells of explants were clearly distinguishable, even after 6 weeks of culture (Fig. 3g). The majority of the epidermal and cortex cells were rich in amyloplasts, which stained intensely with toluidine blue. The axial cylinder appeared to be structurally intact. During the first leaf stage, the somatic embryos showed a normally developed vascular system and the first leaf, shoot apex and the primordium of the second leaf were also visible (Fig. 3g). On some explants, all the somatic embryos produced during the first 6 weeks of culture were almost at the same stage of development (Fig. 3h).

Somatic embryos which developed in darkness were opaque (Fig. 3h) or somewhat translucent at the base (Fig. 3d), but apically, were white, yellow or greenish in color. At transition from embryo to juvenile sporophyte, the lamina of the embryonic leaf became pale green during the next week of culture, and the whole leaf elongated (Fig. 3i). The next step of sporophyte development was the formation of the second frond and the elongation of the root. On returning to light, the frond lamina of the etiolated sporophyte quickly became dark green and regained its typical shape (Fig. 3j).

### The efficiency of somatic embryogenesis in short- and long-term culture

**Fig. 4:**
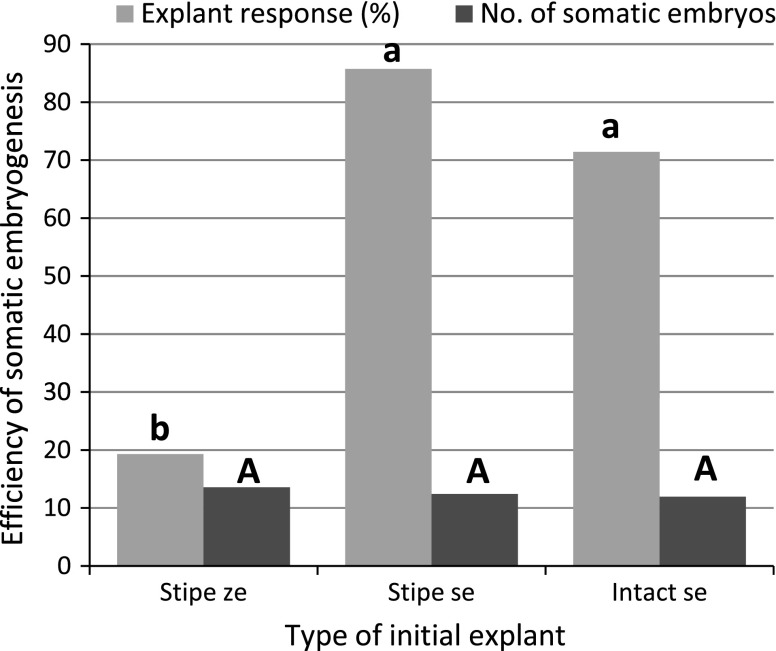
Efficiency of somatic embryogenesis induced for both zygotic and somatic embryo-derived explants following 9 weeks of culture in the dark. Medium 1/2MS supplemented with 2 % (w/v) sucrose. *ze* zygotic embryo developed under photoperiod conditions, *se* somatic embryo developed in darkness

When stipes of the first frond of zygotic embryos were cultured on 1/2MS agar medium containing 2 % sucrose, primary somatic embryos were formed at a frequency of 19.3 % over a 9 week period (Fig. 4). When SE was induced in stipes of somatic embryo-derived juvenile sporophytes, 85.71 % of explants formed new somatic embryos. In intact somatic embryos, the percentage of responding explants was lower (71.43 %), but the difference was statistically insignificant. The average number of somatic embryos produced was similar for each type of initial explant studied, ranging from 11.97 to 13.57 per stipe (Fig. 4).

Somatic embryo-derived stipe explants were able to produce somatic embryos within 11 months of maintenance on 1/2MS medium supplemented with 2 % sucrose without any subculture. The cultures were kept constantly in darkness. The frequency of explants producing somatic embryos ranged between 61.0 and 86.4 % (Table 1). The efficiency of *C.* *delgadii* SE in long-term culture differed significantly (Table 1). After 2 months, each stipe produced 13.2 somatic embryos. The embryogenic potential increased gradually, reaching 84.3 and 80.1 somatic embryos per responding explant by the end of months 9 and 10, respectively. The efficiency of SE decreased to 55.2 embryos per stipe after 11 months of continuous culture. The highest SEPCI index was based on 10-month-old cultures.

**Table 1:** Efficiency of *Cyathea delgadii* somatic embryogenesis during 11 months of culture on 1/2MS medium supplemented with 2 % sucrose, without any subculture, in darkness

Month of culture	Percentage of responding explants	No. of somatic embryos per responding explant	Somatic embryo production capacity index (SEPCI)
2	80.5 ± 3.9ab	13.2 ± 3.6d	10.6
5	74.2 ± 18.2ab	26.1 ± 10.6cd	19.4
6	82.0 ± 19.4ab	34.3 ± 18.2c	28.1
7	61.0 ± 10.4b	54.8 ± 46.4b	33.4
8	61.9 ± 26.1b	58.3 ± 43.8b	36.1
9	68.9 ± 18.5ab	**84.3** ± **44.7a**	**58.1**
10	**86.4** ± **10.8a**	**80.1** ± **53.0a**	**69.2**
11	64.3 ± 14.6b	55.2 ± 44.5b	35.5

Values marked with the same letter do not differ significantly at the 0.05 level according to Fisher’s least significant difference (LSD’s) test. Data represent mean ± standard deviation of three experiments, each consisting of at least 30 explants

Bold values indicate best results

**Fig. 5:**
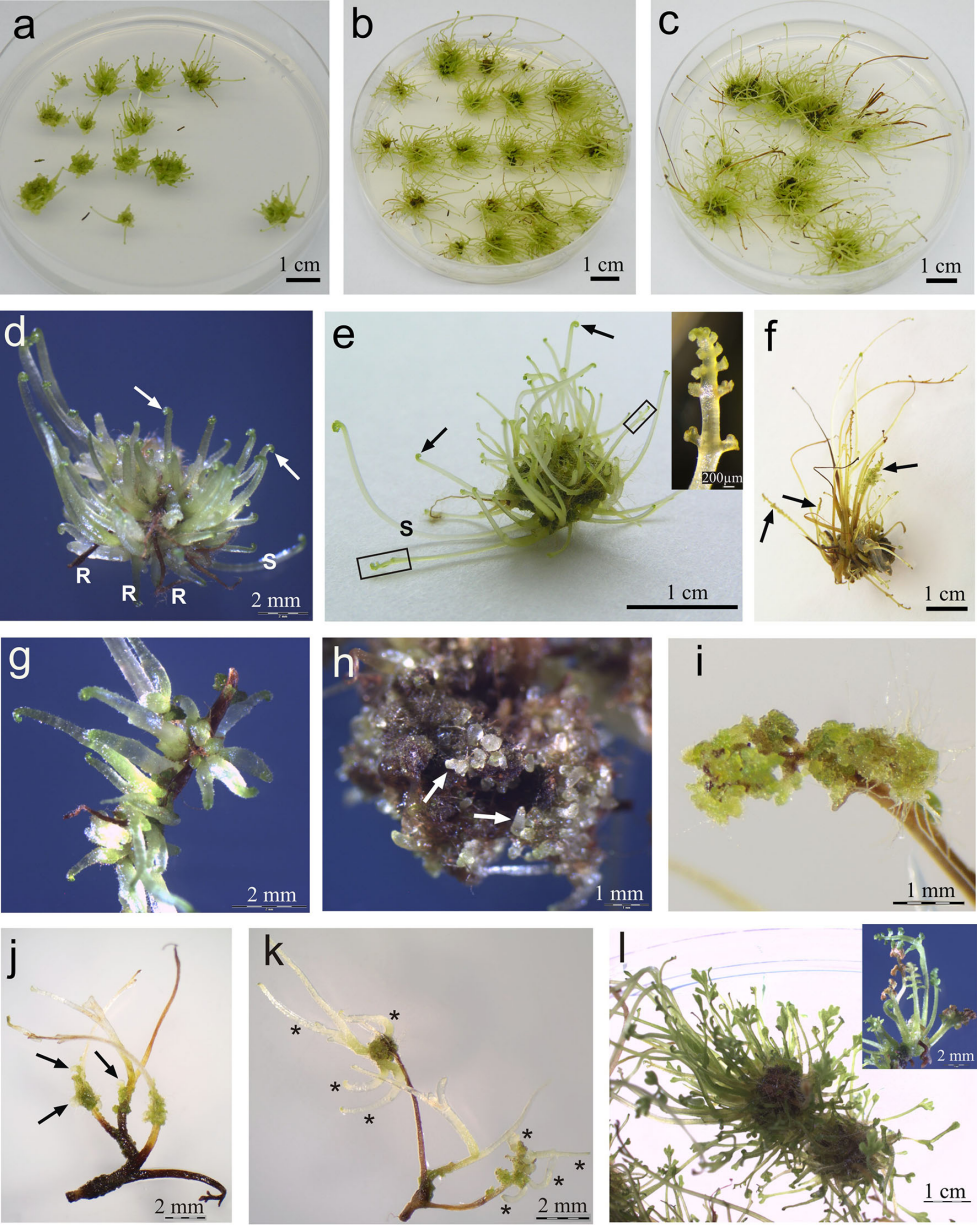
Progress of somatic embryo production in *Cyathea delgadii* during long-term culture. General view of a culture maintained in darkness for: **a** 3 months, **b** 5 months, **c** and 11 months. **d** Numerous young sporophytes with roots and croziers (*arrows*), after 3 months. **e** Typical fronds with long stipes and croziers (*arrows*), with brown tissue at their bases; a few laminae that have developed further (*rectangle* and *small picture*), after 5 months. **f** Spontaneous production of somatic embryos on aging fronds (*arrows*), after 7 months. **g** Further development of spontaneously induced somatic embryos. **h** Somatic embryos derived from the brown tissue (*arrows*), after 8 months. **i** *Yellow* and *green* callus on surface of lamina. **j** *Yellow* and *green* callus on surface of stipes with differentiated somatic embryos (*arrows*). **k** Young sporophytes (*asterisks*) formed by secondary, spontaneous somatic embryogenesis. **l** Image of sporophytes after 2 week-long light exposure. *R* root, *S* stipe (color figure online)

The progress of an 11-month-old culture maintained in darkness is shown in Fig. 5. The fronds of somatic embryo-derived sporophytes gradually elongated, and the mass of tissue was seen to increase (Fig. 5a–c). After 3 months of culture, mass-produced young sporophytes (Fig. 5d) developed two fronds and one or two roots (see Fig. 1b). After 5 months, the fronds possessed long stipes and most of their laminae remained as croziers (Fig. 5e). A few of these commenced further development (Fig. 5e). After 7 months of culture, the first symptoms of sporophyte aging were observed. Some fronds turned brown (Fig. 5f). During 11 months of extended culture, some fronds died (Fig. 5c).

**Fig. 6:**
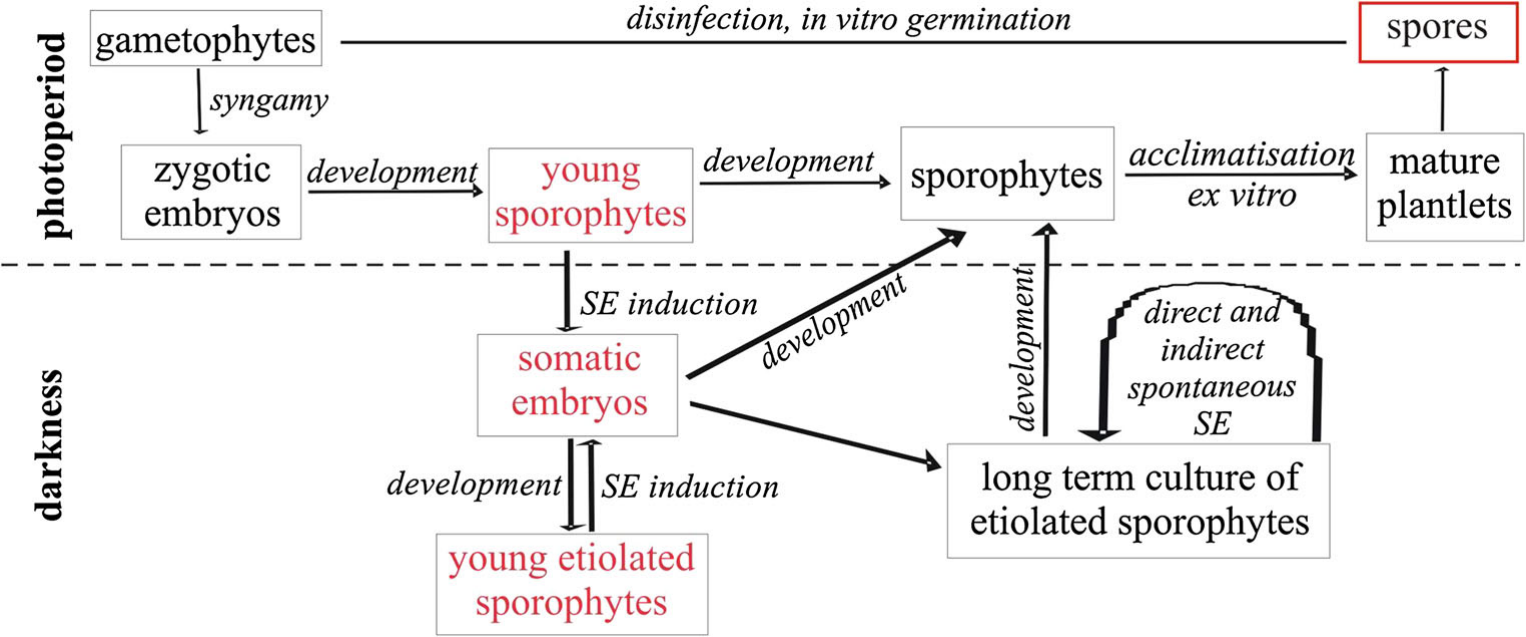
Schematic diagram representing the course of the newly described somatic embryogenesis process for *C.* *delgadii*: commencing with spores, passing through the gametophyte stage, followed by induction of zygotic and somatic embryogenesis and finally, the production of mature plantlets initially grown in vitro and later under ex vitro conditions. *SE* somatic embryogenesis, initial explants are shown in *red* (color figure online)

The aging fronds spontaneously produced new somatic embryos directly on their laminae and stipes (Fig. 5f, g). These somatic embryos were capable of further development without any subculture (Fig. 5g). Alternative ways of embryo formation were also investigated. When cultures were maintained in darkness for at least 5 months, brown tissue was formed at the base of sporophytes (Fig. 5e). During the next 3 months, somatic embryos arose from this tissue (Fig. 5h). Moreover, the laminae and stipes of aging fronds developed embryogenic callus tissue (Fig. 5i, j). The resultant somatic embryos were also able to develop into sporophytes (Fig. 5k). Under 16/8 h photoperiod conditions, all etiolated sporophytes turned green and developed normally to form plantlets (Fig. 5l). Sporophytes began to produce spores after 6 months of growing under ex vitro conditions. The process of SE for *C.* *delgadii* is shown schematically in Fig. 6. It commences with spores, passes through the gametophyte stage, the induction of zygotic and somatic embryogenesis, the growth of mature plantlets and their acclimatization to ex vitro conditions, and ends with a second generation of spores (Fig. 6).

### Plant regeneration and acclimatization

After 3 weeks of culture of initial explants, embryos were formed (Fig. 3a). Most of these had the capacity for further development. Under conditions of constant darkness, embryos developed a few roots and fronds, but the latter remained at the crozier stage for several months (Fig. 5d, e). When a 3-week-old initial culture was subjected to photoperiod conditions, the embryos expanded sequentially and developed into sporophytes without any additional subculture (Fig. 5l). The sporophytes grew quickly and, within 8 months, their acclimatization to ex vitro conditions was complete. Following acclimatization, 23 of the 25 tested sporophytes survived and were transferred to a greenhouse. During the next 6 months, the sporophytes grew to maturity. They produced spores that were able to germinate and form fertile gametophytes (Fig. 6).

## Discussion

In this paper, we report on a novel SE experimental system for ferns. Our aim was to pioneer a new line of research into this process. This we accomplished by investigating a species of the Monilophyta clade. We discovered that epidermal cells of *C. delgadii* stipe explants have the potential to initiate somatic embryo development equal to that of many spermatophytes. However, it is much easier to evoke the acquisition and expression of embryogenic capacity of somatic cells in ferns than in spermatophytes. This study has enabled us to develop an effective, reproducible and rapid method of propagating *C.* *delgadii* sporophytes. Furthermore, the method of cultivation presented here is particularly attractive in that the use of exogenous PGRs is not required.

### Somatic versus zygotic embryogenesis in ferns

Species belonging to Cyatheaceae, like most leptosporangiate ferns, exhibit a type of embryology in which the first zygotic division is transverse to the polar axis of the gametophyte and longitudinal to the axis of the archegonium (Johnson and Renzaglia 2009). According to Wardlaw (1955) this first division is slightly asymmetric in *Pteris serratula* L.f. and *Marsilea*. The following two divisions give rise to a 4-celled zygotic embryo. Contrary to reports of typical leptosporangiate fern zygotic embryogenesis, somatic embryo formation in *C. delgadii* was initiated by several cell divisions perpendicular to the polar axis of the stipe. These divisions led to the formation of a linear somatic pro-embryo. Despite these differences between somatic and zygotic embryogenesis in the way the first division occurs, complete *C. delgadii* somatic embryos were produced. This may strengthen the hypothesis that the initial division may not be important in the fate determination of the embryonic cell in pteridophyte groups (Johnson and Renzaglia 2009). Variations in the plane of zygotic division in leptosporangiate ferns (particularly in angle shift) may be regulated by its proximity to an auxin-secreting meristematic region of the gametophyte, gravity or gametophyte habit (Stone 1958; Jayasekera and Bell 1959). Thus, we speculate that the pattern of early SE is also determined by the impact of the polar gradient of endogenous hormones as the latter pass from the blade to the base of the frond.

In zygotic embryogenesis of leptosporangiate ferns, following the first division of the zygote, the epibasal cell becomes located towards the apex of the prothallus, and after it divides (transversely to the first division), the daughter cells independently give rise to the shoot apex and first leaf. Division of the hypobasal cell leads to the formation of the first root and foot (Wardlaw 1955; Johnson and Renzaglia 2008). Our studies showed that despite the linear course of somatic pro-embryo formation, its further development resulted in division of the embryo body into four clearly visible segments and subsequently into two regions which can be distinguished based on the presence or absence of trichomes. We speculate that the region devoid of trichomes can give rise to foot and embryonic root, whereas the region with trichomes can develop into the shoot apex and embryonic leaf. It seems that despite the lack of an archegonium and adjacent gametophyte tissue able to supply nutrients and growth-regulating substances (e.g. auxins) to the developing zygotic embryo (DeMaggo 1977), the polarity of *C. delgadii* somatic embryos was established very early. This forms the subject of a future investigation.

### Developmental differences between embryo-derived and apogamous sporophyte

The development of *C. delgadii* somatic embryo and juvenile sporophyte was similar to that observed during the zygotic embryogenesis of other leptosporangiate ferns (Wardlaw 1955). Likewise, the first embryonic leaf differed from subsequently formed leaves, and an embryonic root was formed at the base of the first leaf. Development of an apogamous sporophyte differs from the development of an embryo-derived sporophyte in certain details. It was shown that the young apogamous sporophytes of *Asplenium auritum* Sw.*, A. monodon* Liebm., *Phegopteris connectilis* (Michx.) Watt. and *Pteris multifida* Poir. did not form roots in parallel to the first leaf (Kawakami et al. 1995; Soare et al. 2007; Gabancho et al. 2010). Moreover, the first sporophyte leaf possessed stomata, multicellular glandular hairs, and scales (Gabancho et al. 2010). Contrary to the first leaf of the apogamous sporophyte, the juvenile, embryonic leaf of *C. delgadii* did not develop stomata.

### *C. delgadii* somatic embryogenesis versus somatic embryogenesis of seed plants and lycopods

Propagation of spermatophytes in in vitro culture by SE is a complicated process requiring the proper combination of various factors such as: type of plant material, PGRs, sugars, light, mineral salts, etc. (Gaj 2004). For the majority of seed plant species, exogenous PGRs are amongst the most important factors involved in the induction and maintenance of SE (Raemakers et al. 1995; Fehér et al. 2003). Our studies showed that SE in both primary and secondary explants of the fern *C. delgadii* can be induced without the application of PGRs. Moreover, spontaneous formation of somatic embryos and callus production, which improved the effectiveness of the process several fold in 10-month-long culture, occurred without subculturing. This suggests that the juvenile, etiolated stipe explant that was used for culture initiation and a starvation play a crucial role in SE induction in *C. delgadii*. We do not know of any other species for which the process of SE is so highly effective, yet demands so little effort. It is possible that the low margin requirement in the culture may be specific for cryptogamic plants. In the lycopod *Lycopodiella inundata*, the nodular callus and somatic embryo production were established on a MS medium with half-strength mineral salt content, but supplemented with PGRs (Atmane et al. 2000). For a second species of lycopod, *Huperzia selago*, both the induction of callus and the formation of nodular structures on its surface and their subsequent development into somatic embryos occurred on MS hormone-free medium containing an identical mineral salt concentration to that used for *C. delgadii* (Szypuła et al. 2005).

For some species, non-hormonal factors, for example, osmotic shock, drought, mechanical wounding of tissues, macro-salts, heavy metal ions and heat or cold shock, can also be used to induce or enhance SE efficiency (Smith and Krikorian 1989; Choi et al. 1998a; Patnaik et al. 2005; You et al. 2007; Mikuła et al. 2011b). Our study showed that in the case of *C. delgadii*, none of the aforementioned inducers (even mechanical damage) are essential to regain cell totipotency and to acquire the competence necessary to convert somatic cells to embryogenic cells. Our success was probably due to the use of a specific type of explant, i.e. a piece of very young etiolated sporophyte, and maintaining the initial culture in constant darkness. It would appear that endogenous hormone levels in cells and tissues of this species are a major factor in determining cellular response. Choi and Soh (1996) showed that direct somatic embryo formation from ginseng zygotic embryos grown on regulator-free medium was related to the excision of zygotic embryos and polar endogenous auxin accumulation. Conversely, the origin of somatic embryos as multiple or single-state forms, depends on the degree of maturity of the plant material used for experiments (Maheswaran and Williams 1985; Choi et al. 1998b). We have produced somatic embryos of single-cell origin from the maturing epidermis of juvenile sporophyte stipes of C. *delgadii*. According to Maheswaran and Williams (1985), this type of tissue can contain some immature epidermal cells and these single cells are capable of embryogenic response.

### Embryo development

It is well known in pteridophyte embryology that the patterns of zygotic embryo development are defined by the plane of zygotic division and the direction in which the first leaf and shoot apical meristem grow with respect to the gametophyte (Wardlaw 1955; Johnson and Renzaglia 2009). However, in comparison to spermatophytes, the studies of fern embryo development are still incomplete and the developmental stages have not yet been given names. We demonstrated how the pivotal phases of *C. delgadii* somatic embryo formation have morphological counterparts in fern zygotic embryogenesis. Leptosporangiate fern embryos have no suspensor, whereas this is present both in zygotic and most of somatic embryos of seed plants (Young 1995). Instead, during the first stage of fern embryo formation, the foot plays a crucial absorptive and nutritional role in the growth of the embryo (Johnson and Renzaglia 2008). However, using current methods for plant body investigations, we were not able to recognize a foot in the somatic embryos of *C. delgadii*. In future, we intend to use transmission electron microscopy to provide an anatomical framework for studies of somatic embryogenesis.

Another important issue is the designation of morphogenetic stages of the fern embryo, in which the basic structure of the embryo is established. In angiosperms, the development of both zygotic and somatic embryos occurs via the globular, heart, torpedo and cotyledonary stages (Young 1995; Filonova et al. 2000). The sequence of gymnosperm embryo development can be divided into three phases: proembryogeny (up to suspensor elongation), early embryogeny (up to the root meristem establishment) and late embryogeny (including establishment of the root and shoot meristems) (Filonova et al. 2000). At present, we are able only to define three different morphogenetic stages representing three major events in the development of somatic embryo in ferns:

1. linear stage: from the first cell division until the formation of several-celled pro-embryo (Figs. 2, 3a, b);
2. early embryonic leaf stage: until the emergence of the first leaf (Fig. 3c, d);
3. late embryonic leaf stage: until the emergence of the second leaf primordium (Fig. 3e, g, h).

Although the earliest stage of somatic embryo development in *C. delgadii*, comparable to globular stage was not observed, the subsequent developmental patterns appeared to correspond to those observed during the zygotic embryogenesis of leptosporangiate ferns (Wardlaw 1955; Johnson and Renzaglia 2008). The fern zygotic embryo grows and develops its first leaf without any interruption caused by dormancy until it emerges from the gametophytic tissue and becomes established as a free-living sporophyte (Wardlaw 1955). Similarly, the development of the somatic embryo into a juvenile sporophyte is rapid and proceeds directly under the light conditions stated for the tree fern *C. delgadii*.

## Conclusions

Ferns, as a group, are the closest living relatives of spermatophytes, and are of interest not only because of their ornamental value, but also for their usefulness as models for evolutionary, morphological and developmental studies. The phylogenetic position of ferns and their amenability to apogamic reproduction both in vivo and in vitro make them valuable models for studying how asexual plant embryogenesis evolved. The simple, effective and hormone-free system of SE induction described for *C. delgadii* can help broaden our fundamental knowledge of this process. Looking to the future, it also provides an excellent model for the study of endogenous hormonal regulation of SE. This novel method for the in vitro reproduction of tree ferns may also be valuable for the rapid and mass propagation of these plants both for conservation and commercial purposes.

### **Author contribution statement**

The authors have made the following declarations regarding their contributions: Conceived and designed the experiments: AM. Performed the experiments: AM, MP, KT. Analyzed the data: AM, JJR. Contributed reagents/materials/analysis tools: AM. Contributed to the writing of the manuscript: AM, JJR, KT.

## Abbreviations

PGRs: Plant growth regulators
SE: Somatic embryogenesis
1/2MS: Medium containing half-strength Murashige and Skoog micro- and macro-nutrients
